# Efficient heterologous expression of an alkaline lipase and its application in hydrolytic production of free astaxanthin

**DOI:** 10.1186/s13068-018-1180-2

**Published:** 2018-06-27

**Authors:** Jinjin Huang, Zhen Yang, Ruiyan Zhu, Xinxin Qian, Yaqiu Wang, Ying Li, Jilun Li

**Affiliations:** 1 State Key Laboratory of Agrobiotechnology and MOA Key Laboratory of Soil Microbiology, College of Biological Sciences, China Agricultural University, Beijing, 100193 China; 2 Key Laboratory for Biotechnology on Medicinal Plants of Jiangsu Province, School of Life Sciences, Jiangsu Normal University, Xuzhou, 221116 China; 3 Applied Chemistry Key Lab of Hebei Province, Yanshan University, Qinhuangdao, 066004 China

**Keywords:** *Haematococcus pluvialis*, Free astaxanthin, Alkaline lipase, Heterologous expression, Hydrolysis

## Abstract

**Background:**

Astaxanthin, a naturally occurring carotenoid pigment molecule, displays strong antioxidant, anti-cancer, and immunity-enhancing properties, and is often utilized in food, biomedical, cosmetic, and other industries. Free astaxanthin has better solubility than astaxanthin esters (Ast-E), and is a useful auxiliary ingredient in health foods and medicines. Our goal was to establish an improved enzymatic method for preparation of free astaxanthin from natural sources (e.g., the microalga *Haematococcus pluvialis*), to expand the potential applications of free astaxanthin.

**Results:**

The alkaline lipase gene *proalip* and its propeptide were cloned and successfully fusion-expressed in *Pichia pastoris* X-33. The recombinant lipase was termed Lipase-YH. Through optimization of culture conditions (medium formulation, pH, added methanol concentration), cell growth (OD_600_) and secreted enzyme activity respectively reached to 280 and 2050 U/mL in a 50-L autofermentor. Activity of Lipase-YH enzyme powder was about 40,000 U/g. Hydrolysis of Ast-E (extracted from *H. pluvialis*) by Lipase-YH occurred in aqueous phase, and reaction conditions were optimized based on emulsification method and enzyme/substrate ratio. The highest enzymatic reaction rate was observed for substrate concentration 200 μg/mL, with maximal free astaxanthin yield (80%) at 1 h, and maximal Ast-E hydrolysis rate 96%, as confirmed by TLC, HPLC, and mass spectroscopy.

**Conclusion:**

A novel, efficient enzymatic process was developed for production of free astaxanthin through hydrolysis of Ast-E. Lipase activity was enhanced, and production cost was greatly reduced. The unique structure of free astaxanthin allows linkage to various functional compounds, which will facilitate development of novel pharmaceutical and food products in future studies.

**Electronic supplementary material:**

The online version of this article (10.1186/s13068-018-1180-2) contains supplementary material, which is available to authorized users.

## Background

Astaxanthin (3,3′-dihydroxy-β,β-carotene-4,4′-dione) is the principal carotenoid pigment in algae, yeasts, plants, crustaceans, and certain fish (notably salmon) [1–3]. It has molecular formula C_40_H_52_O_4_ and molecular weight 596.86. Astaxanthin has a long conjugated double bond, similar to other carotenoids, but the benzene ring at each end of the carbon chain has a hydroxyl group and a ketone [4]. The unique molecular structure of astaxanthin confers a strong ability to remove oxygen free radicals and to inhibit singlet oxygen [5]. The antioxidant activity of naturally occurring astaxanthin is about 10 times higher than that of β-carotene, and about 100 times higher than that of vitamin E, resulting in the nickname “super vitamin E” [5–7]. Astaxanthin has been found to display anti-cancer, anti-aging, immunity-enhancing, and other beneficial physiological effects in many studies, and is often utilized in the food, biomedical, cosmetic, and animal feed industries [8–12].

The terminal structure of the astaxanthin molecule contains a hydroxyl group that can form an ester linkage with fatty acid to generate astaxanthin esters (Ast-E). Both free astaxanthin and Ast-E are strongly hydrophobic. Addition of certain groups to hydroxyl groups of free astaxanthin can increase solubility, which is useful for preparation of derivatives for oral administration; e.g., derivatives based on astaxanthin disuccinic acid disodium salt have been applied for treatment of various neurological disorders [13]. Derivatives based on free astaxanthin have been successfully used in nutraceutical and medicinal preparations for treatment of degenerative diseases [14].

Antarctic shrimp (*Pandalus borealis*) and microalga *Haematococcus pluvialis* are the richest known sources of natural astaxanthin, which constitutes 2–3% (w/w) of cell dry weight [15–17]. However, free astaxanthin accounts was only 5% (w/w) of total astaxanthin in *H. pluvialis*, while astaxanthin monoester and diester account was, respectively, 70 and 25% [18]. A major problem to be solved for efficient preparation of astaxanthin derivatives is how to convert these large amounts of Ast-E into free astaxanthin.

Nagao’s group used a two-step process involving two types of lipase to produce free astaxanthin [19]. Triglyceride was hydrolyzed by *Candida rugosa* lipase, the by-product fatty acid was removed by molecular distillation, Ast-E was concentrated, and substrate reaction was catalyzed by *Pseudomonas aeruginosa* lipase, resulting in free astaxanthin content 89.3% after 110 h reaction [19]. Ast-E from *Euphausia superba* Dana was hydrolyzed by *C. rugosa* lipase (lipase type VII) and cholesterol esterase from *P. fluorescens*, using bile salts as emulsions to investigate fatty acid composition of astaxanthin [20]. Halldorsson et al. found that lipases from *Pseudomonas*, *Geotrichum candidum*, *Rhizopus delemar*, *R. oryzae* and *Penicillium roqueforti* also functioned as catalysts with the ability to hydrolyze astaxanthin diester to produce monoester; however, only *C. rugosa* lipase produced free astaxanthin, with maximal content (73%) attained after 42 h reaction [21]. The above-described free astaxanthin production processes were all time-consuming and had low conversion rates.

In a 2011 study, we screened various lipases, and observed highest enzyme specificity for a lipase termed ALIP [4]. We obtained free astaxanthin by hydrolysis of total esters extracted from *H. pluvialis* cells by a one-step hydrolysis method, with maximal yield (63.2%) reached after 7 h enzymatic reaction at 25–28 °C [4]. Three problems remained to be solved: (i) ALIP gene was cloned from *Penicillium cyclopium* var. *albus* and expressed in *Pichia pastoris* GS115, but enzyme activity of ALIP needs to be improved; (ii) Buffered complex glycerol/methanol medium (BMGY/BMMY) is a high-cost medium not suitable for extended, large-scale enzyme production; (iii) the grinding method used to emulsify Ast-E is time-consuming, may lead to oxidation of Ast-E, and is not suitable for large-scale production; the reaction process should be completed within a short time.

Our goal in the present study was to improve enzyme activity and increase free astaxanthin yield, to substantially increase astaxanthin solubility for biomedical and nutritional applications. We constructed a recombinant lipase and optimized the enzyme production process and hydrolysis conditions, resulting in significant enhancement of enzyme activity and catalytic efficiency, and reduction of production cost.

## Results

### Enzyme activity enhancement by added propeptide fusion expression

Amino acid (a.a.) sequence of *P. cyclopium* var. *albus* lipase (ALIP) was obtained from GenBank (Seq ID # AAF82375.1), and the signal sequence was predicted by SignalP-4.0 Server program (http://www.cbs.dtu.dk/services/SignalP/). ALIP contained a 20-a.a. signal peptide (Additional file 1: Fig. S1A), and a 7-a.a. propeptide was found at the start of the 258-a.a. mature lipase (Additional file 1: Fig. S1B) [22]. Some propeptides play important roles in folding and secretion of enzymes [23]. We, therefore, investigated the effect of the 7-a.a. propeptide on heterologous expression of ALIP. Lipase gene with propeptide (*proalip*; 795 bp) and without propeptide (*malip*; 774 bp) was obtained by PCR, using *P. cyclopium* var. *albus* cDNA as template. Two gene fragments were each connected to two vectors pPICZαA and pPICMαA [24], resulting in four recombinant plasmids termed pPICZαA-*proalip*, pPICMαA-*proalip*, pPICZαA-*malip*, and pPICMαA-*malip*. Each of these plasmids was transformed to *P. pastoris* X-33 by electroporation, and positive recombinant strains zα-*proalip*-X33, mα-*proalip*-X33, zα-*malip*-X33, and mα-*malip*-X33 were successfully screened.

Four transformants were selected for shake-flask fermentation with BMGY/BMMY, using original strain 9K-*malip*-GS115 [4] as control. Sampling and detection were performed at 24-h intervals. After 168 h, measured enzyme activities of mα-*malip*-X33, zα-*malip*-X33, mα-*proalip*-X33, and zα-*proalip*-X33 were, respectively 12.2–25.4, 36.6–47.5, 238.4–287.3, and 246.2–307.8 U/mL (Table 1). Enzyme activities of mα-*proalip*-X33 and zα-*proalip*-X33 (pro-ALIP) were higher than those of 9K-*malip*-GS115 (133.4 U/mL) and of recombinant without propeptide (12.2–47.5 U/mL).

**Table 1:** Extracellular enzyme activity of various recombinant strains in flask fermentation

Strain	Enzyme activity (U/mL)	Strain	Enzyme activity (U/mL)
mα-*malip*-X33-1	25.4 ± 2.2	mα-*proalip*-X33-1	287.3 ± 22.8
mα-*malip*-X33-2	22.4 ± 3.9	mα-*proalip*-X33-2	238.4 ± 18.9
mα-*malip*-X33-3	12.2 ± 2.5	mα-*proalip*-X33-3	266.5 ± 36.3
mα-*malip*-X33-4	15.3 ± 2.6	mα-*proalip*-X33-4	257.1 ± 42.1
zα-*malip*-X33-1	47.5 ± 7.8	zα-*proalip*-X33-1	307.8 ± 25.5
zα-*malip*-X33-2	42 ± 4.7	zα-*proalip*-X33-2	261 ± 18.2
zα-*malip*-X33-3	45.8 ± 5.6	zα-*proalip*-X33-3	246.2 ± 21.7
zα-*malip*-X33-4	36.6 ± 3.2	zα-*proalip*-X33-4	274.6 ± 43.6
9K-*malip*-GS115	133.4 ± 7.3		

**Fig. 1:**
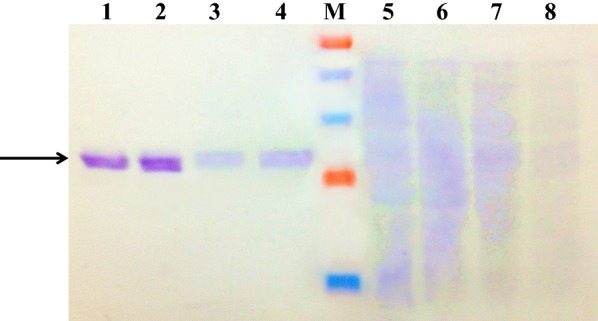
Extracellular and intracellular proteins of four recombinant strains detected by Western blotting. Lanes 1–4: fermentation supernatants of zα-*proalip*-X33-1, mα-*proalip*-X33-1, mα-*malip*-X33-1, and zα-*malip*-X33-1. Lanes 5–8: broken-cell intracellular fluids of the four strains. Lane M: molecular weight markers (100, 60, 45, 28, 18 kDa). Arrow: target protein. Extracellular target proteins proALIP (30 kDa) and mALIP (26 kDa) (arrow) were found in broths of the four strains. Target proteins did not accumulate within cells

Extracellular and intracellular target proteins were detected using identical volumes (32 μL) of fermentation supernatant containing zα-*malip*-X33-1, mα-*malip*-X33-1, zα-*proalip*-X33-1, or mα-*proalip*-X33-1, and identical numbers of cells of the four recombinants. Extracellular target protein pro-ALIP (30 kDa) was detected in zα-*proalip*-X33-1 and mα-*proalip*-X33-1 supernatants (Fig. 1, lanes 1, 2; arrow). mALIP (26 kDa) was found in mα-*malip*-X33-1 and zα-*malip*-X33-1 broths (lanes 3, 4). No target protein band was observed in disrupted cell solution (lanes 5–8), and there was no significant protein accumulation in the four recombinants. These findings indicate that addition of the 7-a.a. propeptide from the start of ALIP promotes protein expression. Secretion was enhanced sixfold than zα-*malip*-X33 (47.5 U/mL). The strain showing highest lipase secretion (307.8 U/mL), produced from recombinant zα-*proalip*-X33-1, was selected for subsequent experiments. This strain was termed X33-YH, and its secreted lipase was termed Lipase-YH.

A comparison of optimal temperature and optimal pH of ALIP with and without the propeptide is shown in Additional file 2: Table S1. Optimal reaction temperature range for ALIP was 35–40 °C, whereas Lipase-YH maintained high enzyme activity in a broader range 30–50 °C. Optimal reaction pH of ALIP (8.0) was higher than that of Lipase-YH (7.5).

### Optimization of culture conditions

**Fig. 2:**
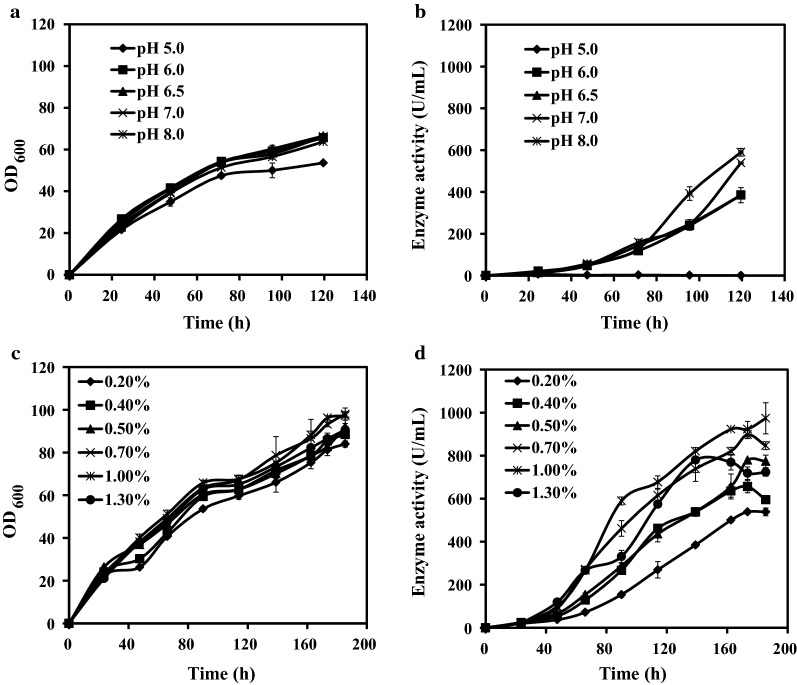
Cell density and extracellular enzyme activity of X33-YH under various culture conditions in flask fermentation. **a** Effect of culture pH on cell density (OD_600_). **b** Effect of pH on enzyme activity. **c** Effect of added methanol concentration on cell density. **d** Effect of added methanol concentration on enzyme activity. pH and methanol concentration had no notable effect on cell density, but did affect enzyme activity. Optimal values for Lipase-YH expression in shake-flask culture were pH 7.0–8.0 and methanol 1.0%; resulting enzyme activity was 972 U/mL

To promote large-scale production of Lipase-YH and reduce associated cost, we optimized fermentation pH, formulation of yeast nitrogen base without a.a. (YNB) (see M&M) (Table 2), and methanol concentration in medium for maximal production. Similar results were obtained for three batches. Effects of fermentation pH and methanol concentration on enzyme activity are shown in Fig. 2, and those of BMMY containing various YNB formulations are shown in Table 2, using averaged data of three repeats from a typical batch.

**Table 2:** Effects of media with various YNB formulations on cell density (OD600) and enzyme activity of X33-YH cultured for 124 h

YNB type	Components of YNB	BMMY medium	OD_600_ (124 h)	Enzyme activity (124 h) (U/mL)
a1-YNB	Nitrogen source, vitamins, trace elements, macronutrients	a1-BMMY	63.34 ± 2.16	623.3 ± 10.8
a2-YNB	Nitrogen source, trace elements, macronutrients	a2-BMMY	63.55 ± 1.94	600.2 ± 15.2
a3-YNB	Macronutrients	a3-BMMY	63.48 ± 1.29	615.6 ± 12.7
c-YNB	With commercial YNB	c-BMMY	64.24 ± 3.15	584.8 ± 21.7
n-YNB	Without YNB	n-BMMY	13.41 ± 1.22	10.8 ± 1.8

Effects of fermentation pH (5.0, 6.0, 6.5, 7.0) on extracellular enzyme activity in shake-flask fermentation were observed. Cell density (OD_600_) and enzyme activity were measured in samples taken at 24-h intervals. Density as a function of time was very similar for pH values in the range 6.0–8.0 (OD_600 _=68 at 120 h), but was notably lower for pH 5.0 (OD_600 _=52 at 120 h) (Fig. 2a). Enzyme activity consistently increased as pH increased from 5.0 to 8.0, and maximal value (577 U/mL at 120 h) was observed for pH 7.0–8.0 (Fig. 2b).

The most expensive component of BMMY is YNB. The formulation of YNB is based on ingredients in four categories: (i) nitrogen source (ammonium sulfate 5.0 g/L); (ii) vitamins (biotin 2 μg/L, folic acid 2 μg/L, calcium pantothenate 400 μg/L, inositol 2 mg/L, *P*-aminobenzoic acid 200 μg/L, thiamin hydrochloride 400 μg/L, riboflavin 200 μg/L, pyridoxine hydrochloride 400 μg/L and nicotinamide 400 μg/L); (iii) trace elements (CuSO_4_ 40 μg/L, FeCl_3_ 200 μg/L, ZnSO_4_ 400 μg/L, MnSO_4_ 400 μg/L, boric acid 500 μg/L, potassium iodide 100 μg/L and sodium molybdate 200 μg/L); (iv) macronutrients (KH_2_PO_4_ 0.85 g/L, K_2_HPO_4_ 0.15 g/L, NaCl 0.1 g/L, CaCl_2_ 0.1 g/L, MgSO_4_ 0.5 g/L, (NH_4_)_2_SO_4_ 5 g/L). In an effort to reduce cost, we tested commercially available YNB (cat # BD291930, Becton Dickinson; Franklin Lakes, NJ, USA), artificial YNB containing all components (termed a1-YNB), artificial YNB without vitamins (termed a2-YNB), and artificial YNB containing only macronutrients (termed a3-YNB) in shake-flask fermentation to determine optimal YNB formulation. The formulations are summarized in Table 2. a1-, a2-, and a3-YNB were added separately to BMMY (mixtures termed a1-BMMY, a2-BMMY, and a3-BMMY). To assess effects of each YNB component on shake-flask Lipase-YH production, we performed concurrent experiments using BMMY with a1-YNB, a2-YNB, a3-YNB, commercial YNB (see M&M) (c-BMMY), and without YNB (n-BMMY). Following addition of 1% methanol, samples were taken at 24-h intervals for measurement of enzyme activity and cell density (OD_600_) (see M&M), and final values were measured at 124 h. Extracellular enzyme activity values were similar for culture of recombinant strain X33-YH in BMMY containing c-YNB (623.3 U/mL), a1-YNB (600.2 U/mL), a2-YNB (615.6 U/mL), and a3-YNB (584.8 U/mL) (Table 2). In striking contrast, the value for culture in medium without YNB (n-YNB) was only 10 U/mL, indicating that macronutrients (but not other components as above) in medium are essential for Lipase-YH production.

We next optimized concentration of added methanol in medium for induction of Lipase-YH production in X33-YH. Methanol concentration was successively increased (0.2, 0.4, 0.5, 0.7, 1.0, 1.3%) by addition at 24-h intervals, and samples were taken for measurement of OD_600_ and enzyme activity. Cell density was not notably affected by alteration of methanol concentration, and highest OD_600_ values were in the 85–90 range (Fig. 2c). Extracellular enzyme activity increased from 568 to 972 U/mL as methanol concentration increased from 0.2 to 1.0%, but then dropped to 788 U/mL when methanol concentration 1.3% (Fig. 2d). We concluded that methanol concentration 1.0% in medium was optimal for inducing Lipase-YH expression. Simultaneous application of the three optimized conditions as above (fermentation pH, YNB formulation, methanol concentration) resulted in increase of enzyme activity from 307.8 to 972 U/mL, with reduction of culture medium cost.

### Enzyme activity 2000 U/mL was attained in 50-L autofermentor

**Fig. 3:**
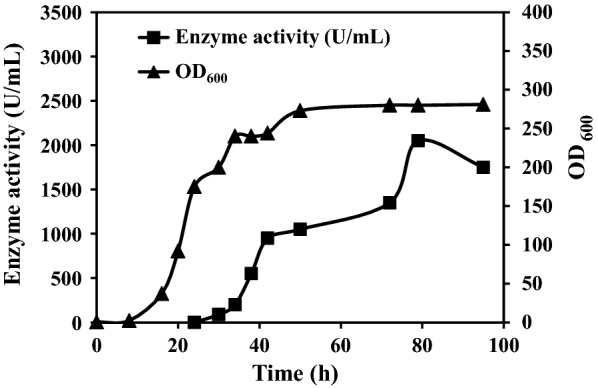
Cell density and extracellular enzyme activity of X33-YH in 50-L autofermentor. Enzyme activity reached 2050 U/mL and cell density (OD_600_) reached 280 after 79 h culture

We next cultured X33-YH in a 50-L autofermentor, using optimized culture conditions as above. The three processing phases were glycerol batch phase, metal ion mixture fed-batch phase, and methanol induction phase (see M&M/“Fermentation in 50-L autofermentor”). Samples were taken at 4-h intervals for measurement of enzyme activity and cell density (OD_600_). Consistent results were obtained in multiple experiments; a representative example is shown in Fig. 3. Maximal OD_600_ value (172) was observed at the end of metal ion mixture fed-batch phase (24 h), when 0.5% methanol was added. Enzyme activity in extracellular increased rapidly between 24 and 79 h, although cell growth was slow. Enzyme activity reached 2050 U/mL, with OD_600_ value 280, at 79 h. In comparison with results using a 7.5-L autofermentor (culture time 96 h, maximal enzyme activity 1575 U/mL) (Additional file 3: Fig. S2), the 50-L autofermentor had significantly lower culture time (79 h) and higher enzyme activity (2050 U/mL).

For preparation of enzyme powder, fermentation broth was centrifuged at 4000 rpm for 5 min at 4 °C, and 150 mL supernatant was added with acetone as precipitant and then dried at room temperature. Similar results were obtained in three repeated experiments, and average values are shown in Additional file 4: Table S2. Optimal supernatant/acetone ratio was 1.0: 2.4 (v/v), 5.05 g enzyme powder was extracted from 150 mL supernatant, and total enzyme activity was 20 × 10^4^ U (Additional file 4: Table S2), which is about 40,000 U/g.

### Preparation of free astaxanthin

**Fig. 4:**
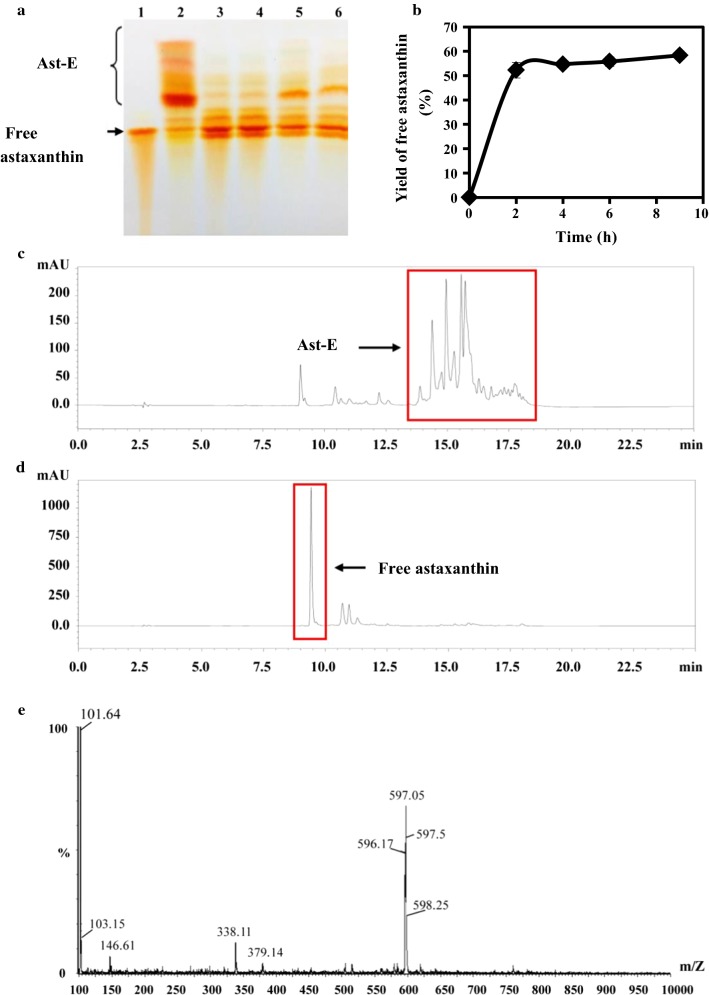
Hydrolysis of Ast-E by Lipase-YH. **a** TLC analysis. Lane 1: free astaxanthin. Lane 2: reaction at 0 h. Lane 3: Lipase-YH produced by X33-YH. Lane 4: pro-ALIP produced by mα-*proalip*-X33-1. Lane 5: mALIP produced by 9K-*malip*-GS115. Lane 6: mALIP produced by zα-*malip*-X33-1. **b** HPLC analysis of Ast-E hydrolysis catalyzed by Lipase-YH enzyme powder dissolved in water. **c** HPLC spectrum at 0 h. **d** HPLC spectrum at 2 h. **e** MS of peak at RT = 16.006 min. Lipase-YH hydrolyzed Ast-E into free astaxanthin, as confirmed by TLC and HPLC. When Lipase-YH enzyme powder dissolved in water was used as catalyst, free astaxanthin yield reached 57.1%

Lipase activity and free astaxanthin production were compared for various recombinant strains. Effects of propeptide on astaxanthin hydrolysis were analyzed using equal amounts of added enzyme. Lipase activity of zα-*malip*-X33-1 fermentation supernatant was only 47 U/mL; therefore, X33-YH, mα-*proalip*-X33-1, and 9K-*malip*-GS115 fermentation supernatants were diluted with distilled water to give the same enzyme activity. Equal amounts of enzyme were added to 10-mL reaction system, Ast-E (about 86 μg) was added to achieve activity level 4.6 U/μg, and the mixture underwent reaction for 7 h at 30 °C. 500-μL samples were taken at 0 and 7 h for TLC analysis. Ast-E content in Lipase-YH catalytic system and enzyme production by mα-*proalip*-X33-1 (Fig. 4a, lanes 3, 4) was less than in zα-*malip*-X33-1 and 9K-*malip*-GS115 systems (lanes 5, 6), and free astaxanthin content was greater. These findings indicate that lipase activity was enhanced by addition of propeptide, thus improving efficiency of catalytic substrates for production of free astaxanthin.

Catalysis of astaxanthin hydrolysis by Lipase-YH enzyme powder dissolved in water was evaluated. Reaction conditions were: substrate (Ast-E) concentration about 25 μg/mL, enzyme concentration 20 U/g substrate in 10 mL sodium phosphate buffer (pH 7.0), 9 h reaction at 30 °C. Samples were taken every 2–3 h and analyzed by HPLC. After 9 h, free astaxanthin content was 57.1%, and Ast-E content was 13.7% (Fig. 4b). Ast-E was abundant at 0 h (Fig. 4c), but a large proportion underwent hydrolysis into free astaxanthin by 2 h (Fig. 4d). The product shown in Fig. 4d was isolated and purified for MS analysis (Fig. 4e), and confirmed as free astaxanthin. These findings demonstrate that the enzyme powder dissolved in water is effective for free astaxanthin production.

### Optimal conditions for free astaxanthin production

To obtain maximal yield of free astaxanthin, enzymatic hydrolysis of Ast-E was performed in 10-mL reaction volume, and reaction conditions (enzyme concentration, buffer concentration, reaction pH, substrate concentration) were optimized. Reaction product was quantitatively analyzed by HPLC. All data mentioned below are averages of three repeats in one of three batches showing similar results.

**Fig. 5:**
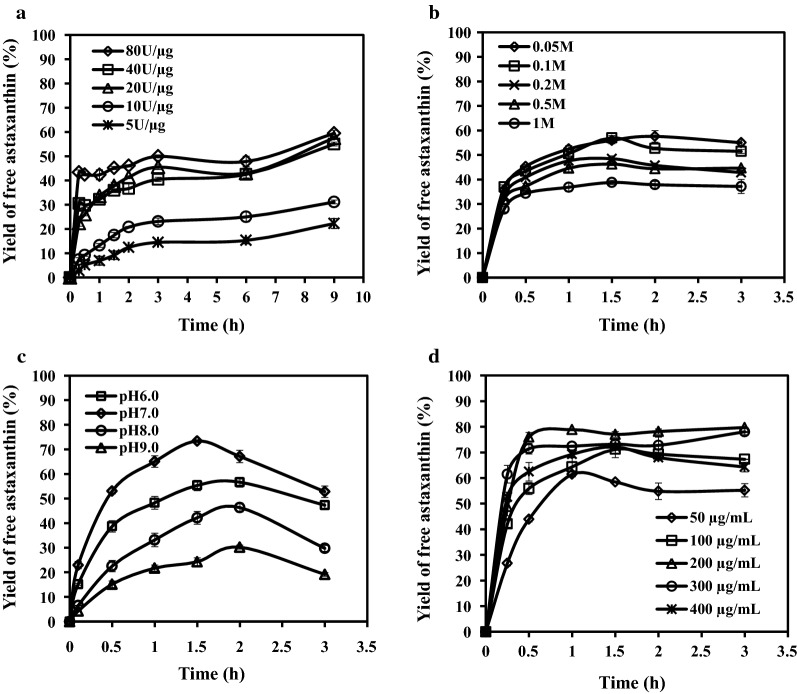
Hydrolysis of Ast-E by Lipase-YH, analyzed by HPLC. **a** Effect of enzyme concentration on free astaxanthin yield. **b** Effect of buffer (sodium phosphate buffer, pH 7.0) concentration on free astaxanthin yield. **c** Effect of pH on free astaxanthin yield. **d** Effect of substrate concentration on free astaxanthin yield. After 1 h culture at pH 7.0, substrate concentration 200 μg/mL, and buffer concentration 0.05 or 0.1 M, enzyme concentration reached 80 U/μg Ast-E, and free astaxanthin yield reached its maximal value (80%)

For each reaction system, substrate concentration was kept constant and various enzyme concentrations (5, 10, 20, 40, 80 U/μg Ast-E) were tested. Enzyme concentration was positively correlated with enzyme reaction speed. For the highest enzyme concentration (80 U/μg Ast-E), free astaxanthin yield increased rapidly to 42% at 15 min, and then more slowly to its maximal value (59%) by 9 h (Fig. 5a).

Effects of various sodium phosphate buffer (pH 7.0) concentrations (0.05, 0.1, 0.2, 0.5, 1.0 M) on free astaxanthin yield were evaluated with other parameters held constant. Samples were taken at intervals and analyzed by HPLC. Free astaxanthin yield was about 56.0% at 1.5 h for buffer concentrations 0.05 and 0.1 M, and less for higher buffer concentrations (Fig. 5b).

In pH experiments, free astaxanthin yield increased as pH rose from 6.0 to 7.0, but declined at higher pH (8.0, 9.0) (Fig. 5c). At pH 7.0, free astaxanthin yield was highest (73.8%) at 1.5 h, and declined for longer durations.

With enzyme concentration kept constant at 80 U/μg Ast-E, effects of various substrate concentrations (25, 50, 100, 200 μg/mL) were investigated. Hydrolytic production rate and free astaxanthin yield increased as substrate concentration increased from 25 to 200 μg/mL (Fig. 5d). For substrate concentration 200 μg/mL, enzymatic reaction was fastest, and free astaxanthin yield was 71.2% after 15 min. Reaction speed declined thereafter, and free astaxanthin yield decreased slightly. Maximal free astaxanthin yield (80.0%) was observed at 1 h, and maximal Ast-E hydrolysis rate was 96%.

Following optimization of reaction conditions as above, we expanded the reaction to 0.5- and 1.0-L systems, and observed free astaxanthin yields and hydrolysis rates similar to those in the 10-mL reaction system. Three batches were performed, with three repeats for each batch. Similar results were obtained for the three batches, and averaged data for one typical batch are shown in Table 3. These findings indicate that optimized conditions for free astaxanthin production we determined in small systems can be successfully extended to larger systems.

**Table 3:** Free astaxanthin yield and Ast-E hydrolysis rate for three reaction systems of increasing volume

Reaction volume	Free astaxanthin yield (%)	Ast-E hydrolysis rate (%)
10 mL	80.0 ± 3.3	96.0 ± 4.3
0.5 L	80.2 ± 5.1	97.0 ± 3.5
1.0 L	79.8 ± 3.8	95.3 ± 3.7

## Discussion

We established an enzymatic process for production of free astaxanthin, a useful compound in the food, biomedical, and cosmetic industries. Enzymatic processes, in comparison with chemical reactions, are generally regarded as efficient, mild, and environmentally friendly. Enzyme activity, enzyme stability, and hydrolysis conditions are key factors in free astaxanthin production and associated cost.

Factors that affect heterologous protein expression include gene structure, signal peptide, gene dosage, propeptide, and culture conditions [24]. We successfully enhanced Lipase-YH production by adding propeptide and optimizing culture conditions. Inouye group demonstrated that a 77-a.a. propeptide organized correct folding of its own protease domain as an intramolecular chaperone, and that Ile to Val (I48V) mutation in the propeptide resulted in production of two subtilisins that differed in secondary structure, thermostability, and substrate specificity [25]. Propeptide sequence played a significant role in in vivo folding and secretion of active *Rhizopus oryzae* lipase (mROL) expressed in *Saccharomyces cerevisiae* [23]. In our 2014 study, addition of propeptide of *Rhizomucor miehei* lipase (RML) increased its expression level in *P. pastoris* from 56 to 430 U/mL, and glycosylation was found in the propeptide [24]. In the present study, NetNGlyc 1.0 server (cbs.dtu.dk/services/NetNGlyc/) predicted no glycosylation following addition of propeptide. We, therefore, hypothesized that the 7-a.a. propeptide at the start of ALIP is involved in enzyme folding and post-translational modification. Protein expression was about sixfold higher for ALIP with vs. without propeptide. Successful enzyme production was achieved using a3-BMMY in a 50-L autofermentor, with enzyme activity > 2000 U/mL, and greatly reduced medium cost (> 70% lower than in previous studies; Additional file 5: Table S3). For culture in a 100-L autofermentor, production time was reduced by about 10 h because of increased oxygen supply. Increase of dissolved oxygen level has been reported to stimulate the methanol utilization pathway and thereby, enhance expression efficiency of heterologous protein expression [26, 27]. Further enhancement of enzyme activity and reduction of culture time in future studies, based on provision of adequate dissolved oxygen, is a strong possibility.

Commercial astaxanthin-based healthcare products use primarily Ast-E derived from *H. pluvialis* or antarctic shrimp (*P. borealis*) extract. Use of free astaxanthin in addition to Ast-E will expand the potential applications of this compound in the nutrition, drug, and food industries. We developed a safe, convenient method of free astaxanthin preparation using Lipase-YH. We optimized various reaction conditions to obtain maximal free astaxanthin yield in a first-generation reaction system. In contrast to methods of enzymatic preparation of free astaxanthin described by Nagao et al. [19], Grynbaum et al. [20], Halldorsson et al. [21], and Zhao et al. [4], we were able to achieve maximal free astaxanthin yield 80% and maximal Ast-E hydrolysis rate 96% with a reaction time of only 1 h. These conditions described here may be further improved in the future. The unique structure of free astaxanthin allows linkage to various functional compounds for development of new drugs, which will expand potential applications [13].

We performed multiple pilot experiments using a small (10 mL) reaction system to conserve substrate, and were able to extend our optimized conditions to larger (0.5, 1.0 L) reaction systems with similar results (Table 3). Subsequent steps in our ongoing studies will involve further optimization of reaction conditions through orthogonal testing, recovery of free astaxanthin from hydrolysis reaction system, and linking to specific functional compounds to further expand astaxanthin applications in various industries as above. Several problems of enzyme and reaction conditions have been resolved in these early-stage experiments. Our upcoming studies will focus on obtaining pure product and linking it to other compounds of interest.

## Conclusion

We constructed a novel recombinant lipase (Lipase-YH), expressed it in *P. pastoris* X-33, and achieved enzyme activity 2050 U/mL through modification of medium formulation and optimization of fermentation conditions. Cost of medium for enzyme production was reduced by > 70%, Ast-E conversion rate reached 96%, and free astaxanthin yield reached 80%. A preliminary attempt was made to efficiently recover free astaxanthin by ultracentrifugation. Our findings provide a basis for future development of new drugs and food products.

## Methods

### Construction of recombinant strains

Mature lipase gene (*malip*) and pro-mature lipase gene (*proalip*) were cloned from *P. cyclopium* var. *albus* (GenBank accession number AF274320.1) cDNA. *malip* was amplified using primer pair *malip*-f (5′-G**GAATTC**GCAACTGCTGACGCCGCT-3′, *Eco*RI site) and *malip*-r (5′-**GCGGCCGC**GCTCAGATAGCCACA-3′, *Not*I site). *proalip* was amplified using primer pair *proalip*-f (5′-CCCG**GAATTC**GCACCTATTTTGGAG TCGA-3′, *Eco*RI site) and *proalip*-r (5′-ATAAT**GCGGCCGC**GCTCAGA TAGCCAC-3′, *Not*I site). Vectors used were pPICZαA (Thermo Fisher Scientific; Waltham, MA, USA) and pPICMαA. pPICMαA was derived from pPICZαA, and α-factor codons of pPICMαA were optimized without change of pPICZαA a.a. sequence [24]. Recombinant plasmids pPICZa-*malip*, pPICMa-*malip*, pPICZa-*proalip*, and pPICMa-*proalip* were constructed as described previously [28]. Plasmids and strains used in this study are listed in Table 4. Four recombinant plasmids (pPICZa-*malip*, pPICMa-*malip*, pPICZa-*proalip*, pPICMa-*proalip*) were linearized by *Bst*X I, and transformed, respectively, to *P. pastoris* X-33 by electroporation. Electroporation and screening of target transformants were performed using an EasySelect *Pichia* expression kit (Thermo Fisher Scientific; Waltham, MA, USA) as per the manufacturer’s instructions.

**Table 4:** Plasmids and strains used in this study

Plasmid or strain	Description	Source
Plasmids		
pPICZαA	Secretion expression vector with α-factor from *S. cerevisiae*	[24]
pPICMαA	pPICZαA with α-factor optimized codons	[24]
pPICZαA-*malip*	pPICZαA ligated with *malip* gene	This study
pPICZαA-*proalip*	pPICZαA ligated with *proalip* gene	This study
pPICMαA-*malip*	pPICMαA ligated with *malip* gene	This study
pPICMαA-*proalip*	pPICMαA ligated with *proalip* gene	This study
Strains		
X-33	Host strain (WT Mut^+^)	[24]
zα-*malip*-X33	*malip* of lipase without propeptide expressed in X-33 using pPICZαA	This study
zα-*proalip*-X33	*proalip* of lipase with propeptide expressed in X-33 using pPICZαA	This study
mα-*malip*-X33	*malip* expressed in X-33 using pPICMαA	This study
mα-*proalip*-X33	*proalip* expressed in X-33 using pPICMαA	This study

9K-*malip*-GS115 was constructed as described by Guan et al. [29]. *malip* was amplified using primer pair as above and connected to pPIC9K to construct recombinant plasmid pPIC9K-*malip*, using Kan screen and gene sequencing (Thermo Fisher Scientific; Waltham, MA, USA). The recombinant plasmid was linearized by *Sal*I and electroporated into *P. pastoris* GS115. Electroporation and screening of target transformants were performed using Multi-Copy *Pichia* Expression Kit (Thermo Fisher Scientific; Waltham, MA, USA) as per the manufacturer’s instructions.

### Optimization of flask fermentation conditions

Flask culture of *P. pastoris* was performed using BMGY [yeast extract 1% (g/v), peptone 2% (g/v), glycerin 1% (g/v)]/BMMY [yeast extract 1% (g/v), peptone 2% (g/v), 100 mM potassium phosphate buffer] medium as described by Hu et al. [30]. Various fermentation conditions were optimized as described below.

#### Formulation of YNB compounds

Formulation of yeast nitrogen base without a.a. (YNB) (cat # BD291930; Becton Dickinson, Franklin Lakes, NJ, USA) was analyzed in terms of four parts: nitrogen source, vitamins, trace elements, and macronutrients. a1-YNB, a2-YNB, and a3-YNB (formulations shown in Table 2) were prepared artificially to retain necessary ingredients and reduce fermentation cost. They were added to BMMY medium (mixtures termed, respectively, a1-BMMY, a2-BMMY, a3-BMMY), rather than commercial YNB, for shake-flask fermentation to determine optimal YNB formulation.

#### Methanol induction

BMGY was used for liquid seed culture, and a3-BMMY [yeast extract 1% (g/v), peptone 2% (g/v), 100 mM potassium phosphate buffer (pH 7.0), a3-YNB 1.34% (g/v)] was used to induce expression of target protein in flask. Increasing concentrations of methanol (0.2, 0.4, 0.5, 0.7, 1.0, 1.3%) in a3-BMMY were established by addition at 24-h intervals, to determine optimal amount of added methanol.

#### Optimal fermentation pH

pH of 100 mM potassium phosphate buffer in a3-BMMY was adjusted to 5.0, 6.0, 6.5, 7.0, and 8.0.

### Fermentation in 50-L autofermentor

Fermentation method for 50-L autofermentor was similar to that in our previous study [31], except that primary seed was cultured in 300 mL BMGY and transferred into 3 L BMGY as second seed. Second seed was then fed into 30 L a3-BMMY in a 50-L autofermentor (Guoqiang; Shanghai, China) as 10% inoculum. The three processing phases were: (i) glycerol batch phase; (ii) metal ion mixture (KH_2_PO_4_/K_2_HPO_4_ 0.85 g/0.15 g, NaCl 0.1 g, CaCl_2_ 0.1 g, MgSO_4_ 0.5 g, glycerol 50% w/v) fed-batch phase; (iii) methanol induction phase. Temperature was kept at 28 °C for phases i and ii, and reduced to 24 °C when target protein was induced by methanol (phase iii). pH was maintained at 7.0 (auto-adjusted by NH_4_·H_2_O) throughout the fermentation process. Agitation rate was 700 rpm, and aeration rate was 10 L/min. When initial glycerol was exhausted, metal ion mixture was fed (feed rate 18.15 mL/h/L initial fermentation volume) for 4 h until glycerol was completely consumed, then intermittently for 1 h. Methanol (0.5% v/v) was added to induce protein expression. Samples were taken at 4-h intervals for measurement of cell density (OD_600_) and secreted enzyme activity.

### Enzyme detection

Lipase activity was detected by NaOH titration as described previously [24]. A reaction mixture consisting of 5-mL olive oil emulsion, 4 mL of 0.1 M HEPES, pH 7.5, and 1.0 mL supernatant was shaken (180 rpm) for 10 min at 50 °C. Reaction was stopped by addition of 15 mL ethanol. Phenolphthalein was added to the mixture as indicator and free fatty acids (FFAs) were titrated with 0.05 M NaOH. One unit (U) lipase activity was defined as the amount of enzyme catalyzing production of 1 μmol FFAs/min [24].

### Detection of target lipase protein by Western blotting

Extracellular pro-ALIP was detected in 40-μL volumes of fermentation supernatant mixture (32 μL fermentation supernatant mixed with 8 μL 5× loading buffer, boiled for 10 min). For each recombinant strain, fermentation broth was centrifuged, supernatant removed, precipitate washed three times and diluted with sterile distilled water to a defined cell density (OD_600 _ = 1.0), and 32 μL diluted cells mixed with 8 μL 5× loading buffer and boiled for 10 min. Intracellular protein content of each strain was determined using 20 μL of mixture as above. Stacking gel (5%), resolving gel (12%), primary antibody, secondary antibody, and BCIP/NBT Chromogenic reagent kit (cat # PA111, Tiangen Biotech; Beijing, China) were as described previously [24].

### Enzyme powder preparation

Enzyme powder was prepared as described by Geethanjali et al. [32]. Pre-cooled acetone was added to 150 mL fermentation supernatant (volume ratio 1.0:0.8–1.0:1.2), precipitated for 1 h at − 20 °C, and dried at room temperature to constant weight. Enzyme powder was cold-stored and diluted to various appropriate concentrations prior to hydrolysis reactions as below. Effects of acetone concentration on enzyme powder extracted from 150 mL Lipase-YH fermentation supernatant were evaluated in terms of four parameters: weight of enzyme powder (dry) (g), total enzyme activity (×10^4^ U), enzyme activity per g (×10^4^ U), recovery rate (%). These parameters were calculated as follows:

- weight of enzyme powder (g): weight of dry enzyme powder obtained from 150 mL Lipase-YH fermentation supernatant.
- total enzyme activity (×10^4^ U): total enzyme activity of all enzyme powder from 150 mL Lipase-YH fermentation supernatant.
- enzyme activity per g (×10^4^ U/g): enzyme activity of 1 g dry enzyme powder.
- recovery rate (%): total enzyme activity (b) divided by total enzyme activity of 150 mL Lipase-YH fermentation supernatant without preparation of enzyme powder.

### Hydrolysis of Ast-E

#### Substrate emulsion

20 mg *H. pluvialis* extract (Ast-E; Jingzhou Natural Astaxanthin Inc., China) was mixed with 20 mg Tween 80. The mixture was dissolved in 5 mL acetone in a 50-mL stoppered flask and dried by nitrogen stream.

#### Hydrolysis reaction system

The above emulsion was added with 10-mL sodium phosphate buffer (0.1 M, pH 7.0), mixed with 40 U/μg (total carotenoids) lipase, and the flask was placed for in a water bath (30 °C, 180 rpm) for 9 h. 300 μL samples were taken at time 0 and at 30-min intervals thereafter. Each sample was mixed with 500 μL acetone and 300 μL *n*-hexane, and centrifuged at 12,000 rpm for 2 min.

#### Optimization of hydrolysis conditions

Optimal values were determined (single-factor optimization technique) for enzyme amount (5, 10, 20, 40, 80 U/μg), reaction buffer concentration (0.05, 0.1, 0.2, 0.5, 1 M), reaction buffer pH (6, 7, 8, 9), and substrate concentration (50, 100, 200, 300, 400 mg/mL). An optimized small hydrolysis system was established according to proportion of each component, and then expanded to 500 mL and 1.0 L.

### Detection of free astaxanthin by TLC, HPLC, and MS

Astaxanthin pigment was detected by TLC on activated silica plates (Silica Gel 60, 10 × 10 cm, thickness 0.2 mm; Yantai Chemical Industry Research Institute; Yantai, China) as we described previously [4]. Reaction supernatant (60 μL) was subjected to HPLC analysis using a CBM-20A system equipped with SPD-M20A diode array detector (Shimadzu; Kyoto, Japan), with sample preparation and test conditions as described by Zhao et al. [4]. Mass spectroscopic (MS) analysis (LCQ Deca XP, Thermo Finnigan; San Jose, CA, USA) of free astaxanthin gave mass-to-charge ratio (m/z) of its parent ion. MS conditions were: capillary temperature 300 °C, spray voltage 4.5 kV, positive ionization, and scan range 100–1000 m/z, as described by Zhao et al. [4].

### Free astaxanthin calculation

3.0 mg astaxanthin standard was dissolved in 10 mL chloroform in a 100-mL volumetric flask in ultrasonic water bath, and volume adjusted to 100 mL with *n*-hexane to obtain stock solution. Various amounts (5, 10, 15, 20 mL) of stock solution were mixed with 4 mL chloroform in 100-mL flasks, and volume adjusted to 100 mL with hexane to obtain four concentrations (1.5, 3.0, 4.5, 6.0 μg/mL) of astaxanthin standard solution. These solutions were measured by HPLC, and an astaxanthin standard curve was constructed relating peak area to quantity of free astaxanthin. Formulas used were:

$${\text{Yield of free astaxanthin }}\left( \% \right)\, = \,{{{{M}}_{\text{free astaxanthin}} } \mathord{\left/ {\vphantom {{{{M}}_{\text{free astaxanthin}} } {{{M}}_{{H. \, pluvialis\,{\text{extract}}}} }}} \right. \kern-0pt} {{{M}}_{{H. \, pluvialis\,{\text{extract}}}} }}$$

$${\text{Hydrolysis rate of Ast-E }}\left( \% \right)\, = \,{{{{A}}_{\text{free astaxanthin}} } \mathord{\left/ {\vphantom {{{{A}}_{\text{free astaxanthin}} } {{{A}}_{\text{Ast-E}} }}} \right. \kern-0pt} {{{A}}_{\text{Ast-E}} }}$$

where *M*_free astaxanthin_ = quantity of free astaxanthin in reaction mixture (computed from standard curve); *M*_*H. pluvialis* extract_ = weight of *H. pluvialis extract* added to reaction mixture at 0 h; *A*_free astaxanthin_ = peak area of free astaxanthin; *A*_Ast-E_ = summed peak area of Ast-E.

## Additional files

**Additional file 1: Fig. S1.** Analysis of *Penicillium cyclopium* var. *albus* lipase gene. **A:** Signal peptide analysis of the gene by signalP-4.0 Server program. **B:** Signal peptide and propeptide of the gene. A 20-a.a. signal peptide and 7-a.a. propeptide were found at the start of the 258-a.a. mature lipase.

**Additional file 2: Table S1.** Comparison of properties of ALIP with and without propeptide (Lipase-YH).

**Additional file 3: Fig. S2.** Culture of X33-YH in 7.5-L autofermentor. X33-YH was cultured in a 7.5-L autofermentor following optimization of culture conditions and medium formulation as described in the text. The three processing phases were glycerol batch phase, metal ion mixture fed-batch phase, and methanol induction phase. Samples were taken at 4-h intervals, and cell density (OD_600_) and enzyme activity were measured. Cell density reached OD_600_ = 251 at the end of glycerol batch phase (43 h), and methanol 0.5% was added at 44 h. Enzyme production rate increased rapidly between 45 and 60 h, although cell growth was slow. Enzyme activity was 1125.7 U/mL at 60 h. Metal ion mixture was added at 57 h. Enzyme production rate declined abruptly after 60 h, and cell density was fairly constant from 60 to 90 h. At 96 h, enzyme activity reached its maximal value (1575 U/mL), and OD_600_ was 311.

**Additional file 4: Table S2.** Effects of acetone concentration on Lipase-YH extraction.

**Additional file 5: Table S3.** Comparison of cost of medium before and after using a3-YNB.

## Abbreviations

a.a.: amino acid(s)
MS: mass spectroscopy
BMGY/BMMY: buffered complex glycerol/methanol medium
YNB: yeast nitrogen base without amino acids
Ast-E: astaxanthin esters
