# Supplementary material for: Efficient heterologous expression of an alkaline lipase and its application in hydrolytic production of free astaxanthin
Source: Biotechnol Biofuels. 2018 Jun 27;11:181. doi: 10.1186/s13068-018-1180-2 (PMC6020301; doi:10.1186/s13068-018-1180-2)
Supplement: Supplementary file 1 — Additional file 1: Fig. S1. Analysis of Penicillium cyclopium var. albus lipase gene. A: Signal peptide analysis of the gene by signalP-4.0 Server program. B: Signal peptide and propeptide of the gene. A 20-a.a. signal peptide and 7-a.a. propeptide were found at the start of the 258-a.a. mature lipase. [file 13068_2018_1180_MOESM1_ESM.pdf]

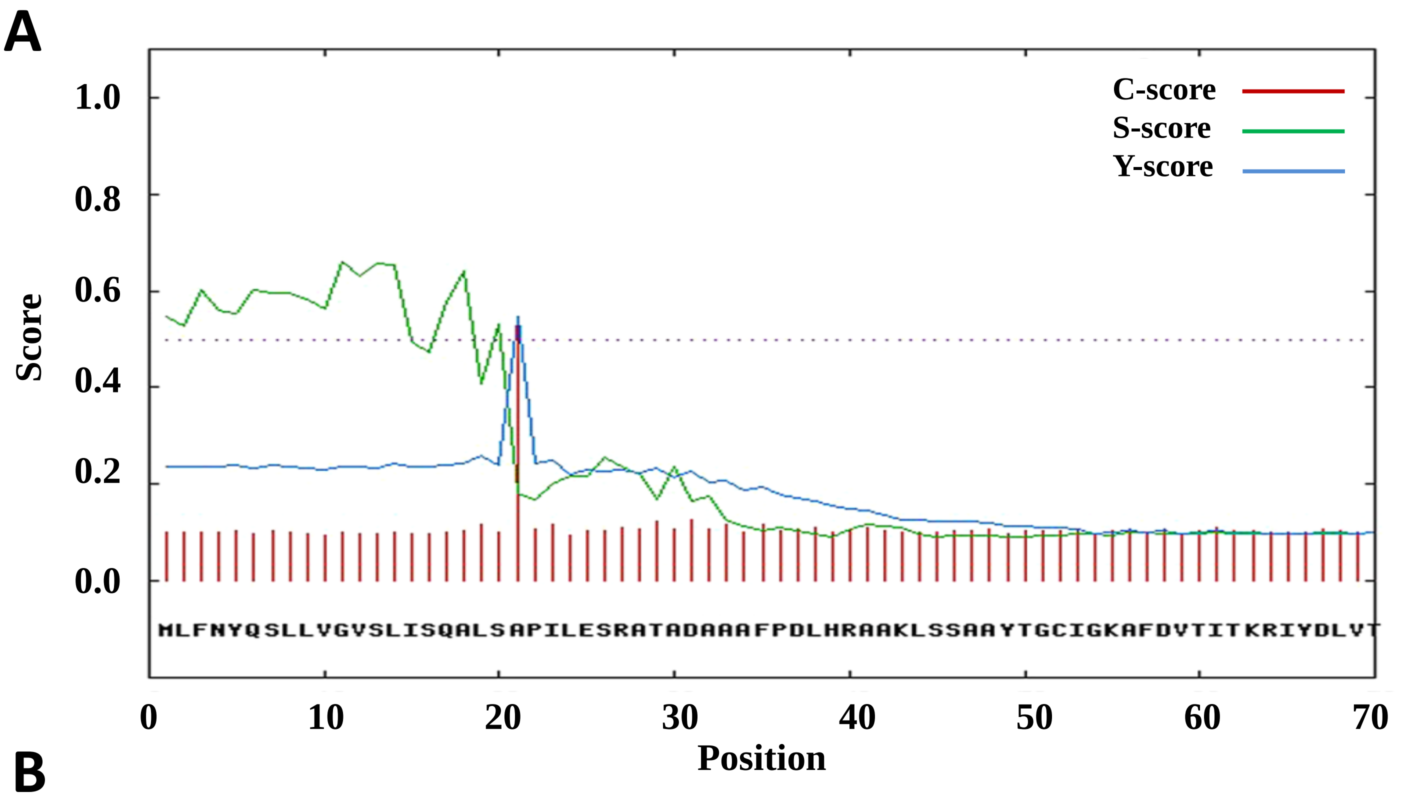

B

```
1    MLFNYQSLLV GVSLISQALS APILESRATA DAAAFPDLHR AAKLSSAAYT GCIGKAFDVT
61   ITKRIYDLVT DTNGFVGYST EKKTIAVIMR GSTTITDFVN DIDIALITPE LSGVTFPSDV
121  KIMRGVHRPW SAVHDTIITE VKALIAKYPD YTLEAVGHSL GGALTSIAHV ALAQNFPDKS
181  LVSNALNAFP IGNQAWADFG TAQAGTFNRG NNVLDGVPNM YSSPLVNFKH YGTEYYSSGT
241  EASTVKCEGQ RDKSCSAGNG MYAVTPGHIA SFGVVMLTAG CGYLS
```

signal peptides   propeptide   mature lipase
