# Supplementary material for: Efficient heterologous expression of an alkaline lipase and its application in hydrolytic production of free astaxanthin
Source: Biotechnol Biofuels. 2018 Jun 27;11:181. doi: 10.1186/s13068-018-1180-2 (PMC6020301; doi:10.1186/s13068-018-1180-2)
Supplement: Supplementary file 2 — Additional file 2: Table S1. Comparison of properties of ALIP with and without propeptide (Lipase-YH). [file 13068_2018_1180_MOESM2_ESM.docx]

**Additional file 2: Table S1.** Comparison of properties of ALIP with and without propeptide (Lipase-YH).

|  | Optimal reaction temperature | Optimal pH |
| --- | --- | --- |
| ALIP | 35-40 °C | 8.0 |
| Lipase-YH | 30-50 °C | 7.5 |
