# Supplementary figures and images for: Efficient heterologous expression of an alkaline lipase and its application in hydrolytic production of free astaxanthin
Source: Biotechnol Biofuels. 2018 Jun 27;11:181. doi: 10.1186/s13068-018-1180-2 (PMC6020301; doi:10.1186/s13068-018-1180-2)

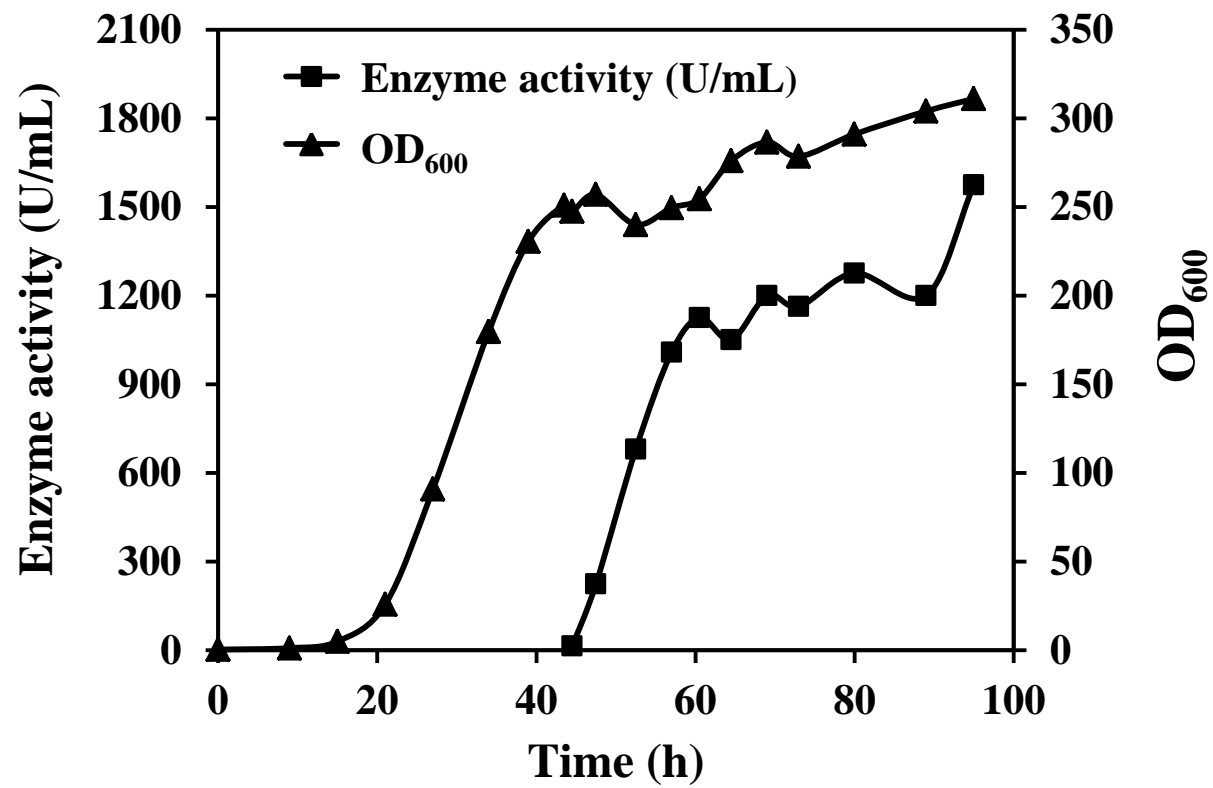

Supplement: Supplementary file 3 — Additional file 3: Fig. S2. Culture of X33-YH in 7.5-L autofermentor. X33-YH was cultured in a 7.5-L autofermentor following optimization of culture conditions and medium formulation as described in the text. The three processing phases were glycerol batch phase, metal ion mixture fed-batch phase, and methanol induction phase. Samples were taken at 4-h intervals, and cell density (OD600) and enzyme activity were measured. Cell density reached OD600 = 251 at the end of glycerol batch phase (43 h), and methanol 0.5% was added at 44 h. Enzyme production rate increased rapidly between 45 and 60 h, although cell growth was slow. Enzyme activity was 1125.7 U/mL at 60 h. Metal ion mixture was added at 57 h. Enzyme production rate declined abruptly after 60 h, and cell density was fairly constant from 60 to 90 h. At 96 h, enzyme activity reached its maximal value (1575 U/mL), and OD600 was 311. [file 13068_2018_1180_MOESM3_ESM.pdf]
