# Supplementary material for: Efficient heterologous expression of an alkaline lipase and its application in hydrolytic production of free astaxanthin
Source: Biotechnol Biofuels. 2018 Jun 27;11:181. doi: 10.1186/s13068-018-1180-2 (PMC6020301; doi:10.1186/s13068-018-1180-2)
Supplement: Supplementary file 4 — Additional file 4: Table S2. Effects of acetone concentration on Lipase-YH extraction. [file 13068_2018_1180_MOESM4_ESM.docx]

**Additional file 4: Table S2.** Effects of acetone concentration on Lipase YH extraction.

| Fermentation supernatant/ acetone volume ratio | Weight of enzyme powder (dry)  (g) | Total enzyme activity  (x 10^4^ U) | Enzyme activity per g (x 10^4^ U/g) | Recovery rate  (%) |
| --- | --- | --- | --- | --- |
| 1.0: 0.4 | 2.4 | 1.11 | 0.46 | 5.21 |
| 1.0: 0.8 | 3.02 | 1.57 | 0.49 | 7.36 |
| 1.0: 1.2 | 3.09 | 1.68 | 0.54 | 7.86 |
| 1.0: 1.6 | 3.57 | 6.13 | 1.72 | 28.67 |
| 1.0: 2.0 | 4.19 | 17.48 | 4.17 | 81.75 |
| 1.0: 2.4 | 5.05 | 20.01 | 3.96 | 93.57 |
| 1.0: 2.8 | 5.16 | 20.00 | 3.88 | 78.39 |
| 1.0: 3.2 | 5.43 | 17.92 | 3.30 | 70.24 |

All data were calculated based on enzyme powder obtained from 150 mL Lipase-YH fermentation broth.
