# Supplementary material for: Efficient heterologous expression of an alkaline lipase and its application in hydrolytic production of free astaxanthin
Source: Biotechnol Biofuels. 2018 Jun 27;11:181. doi: 10.1186/s13068-018-1180-2 (PMC6020301; doi:10.1186/s13068-018-1180-2)
Supplement: Supplementary file 5 — Additional file 5: Table S3. Comparison of cost of medium before and after using a3-YNB. [file 13068_2018_1180_MOESM5_ESM.docx]

**Additional file 5: Table S3.** Comparison of cost of medium before and after using a3-YNB.

|  | Composition | Price  (RMB^a^/kg) | Content of YNB in 1 L BMMY (g/L) | Cost of YNB in 1 L BMMY (RMB/L) | | Cost of 1 L BMMY (RMB/L) | Cost of 1 L BMMY containing YNB (RMB/L) |
| --- | --- | --- | --- | --- | --- | --- | --- |
| c-YNB | YNB | 2800 | 13.4g | 37.52 | 37.52 | 5.56 | 43.08 |
| a3-YNB | KH_2_PO_4_ | 9 | 0.85g | 0.00765 | 0.02803 |  | 5.59  (reduced by 87%) |
|  | K_2_HPO_4_ | 9 | 0.15g | 0.00135 |  |  |  |
|  | NaCl | 2.3 | 0.1 g | 0.00023 |  |  |  |
|  | CaCl_2_ | 8 | 0.1 g | 0.0008 |  |  |  |
|  | MgSO_4_ | 6 | 0.5 g | 0.003 |  |  |  |
|  | (NH_4_)_2_SO_4_ | 3 | 5 g | 0.015 |  |  |  |

^a^ RMB: Chinese Yuan.
